# Comparison of Bilateral Versus Unilateral Transversus Abdominis Plane Block Combined with Spinal Anesthesia in Laparoscopic Appendectomy: A Retrospective Observational Study

**DOI:** 10.3390/diagnostics15172122

**Published:** 2025-08-22

**Authors:** Abdulhakim Şengel, Evren Büyükfırat, Selçuk Seçilmiş, Nuray Altay, Ahmet Atlas, Mahmut Alp Karahan

**Affiliations:** 1 Department of Anesthesiology and Reanimation, Faculty of Medicine, Harran University, Osmanbey Campus, 63200 Sanlıurfa, Turkey; evrenbf@gmail.com (E.B.); nurayaltay@ymail.com (N.A.); ahmetatlas@harran.edu.tr (A.A.); 2 Department of Anesthesiology and Reanimation, Kahta State Hospital, 02600 Adıyaman, Turkey; sleon_02@hotmail.com; 3 Department of Anesthesiology and Reanimation, Şanlıurfa Mehmet Akif Inan Training and Research Hospital, Haliliye, 63040 Sanlıurfa, Turkey; mahmutalp_k@yahoo.com

**Keywords:** analgesia, laparoscopic appendectomy, postoperative pain, regional anesthesia, spinal anesthesia, transverse abdominal plane block

## Abstract

**Background/Objectives:** Laparoscopic appendectomy (LsA) is a standard acute surgical procedure typically performed under general anesthesia (GA). However, GA is associated with side effects such as hemodynamic instability and postoperative nausea/vomiting. Regional anesthesia (RA) has gained attention as an effective alternative in such surgeries, as it reduces surgical stress responses, provides adequate postoperative analgesia, and promotes early mobilization. This study evaluates the effectiveness of the combined use of spinal anesthesia (SA) and transversus abdominis plane block (TAPB) in LsA procedures. **Methods:** This retrospective observational study included 220 patients who underwent LsA between 2020 and 2023. Patients were divided into two groups: Group 1 (*n* = 110) received bilateral TAPB, and Group 2 (*n* = 110) received unilateral TAPB, both under SA. Postoperative pain was assessed using the Visual Analog Scale (VAS), and outcomes such as time to first analgesic requirement, analgesic consumption, and patient satisfaction were recorded. **Results:** This study evaluated the effects of SA combined with TAPB in LsA. Bilateral TAPB significantly prolonged the time to first analgesic request (13.7 vs. 12.1 h; *p* = 0.001) and reduced analgesic requirements (*p* = 0.008) compared to unilateral TAPB. VAS scores were significantly lower in Group 1 at the 9th and 12th hours postoperatively (*p* = 0.003 and *p* = 0.039). Although overall satisfaction scores were similar, a higher proportion of patients in Group 1 reported being “very satisfied” or “excellent” (55.5% vs. 42.7%). **Conclusions:** The combination of spinal anesthesia and bilateral TAPB is a safe and effective anesthetic strategy for LsA. Compared to unilateral TAPB, it offers superior postoperative analgesia and improved patient satisfaction.

## 1. Introduction

Laparoscopic appendectomy (LsA) has become the gold standard for the surgical treatment of acute appendicitis due to its favorable outcomes, including reduced postoperative pain, lower wound infection rates, and shorter hospital stays than open appendectomy [1]. While general anesthesia (GA) remains the most commonly used anesthetic approach in LsA, it is associated with certain drawbacks, such as postoperative nausea and vomiting, hemodynamic fluctuations, and delayed recovery, particularly in patients with high anesthetic risk or comorbidities [2].

Spinal anesthesia (SA), a form of regional anesthesia (RA), has been proposed as an effective alternative to GA in selected laparoscopic procedures. It offers several advantages, including avoidance of airway instrumentation, attenuation of the surgical stress response, and prolonged postoperative analgesia [3]. SA has been successfully applied in laparoscopic cholecystectomy and hernia repair [4,5], and emerging evidence supports its feasibility in LsA [6].

To enhance the analgesic benefits of SA, peripheral nerve blocks such as the transversus abdominis plane block (TAPB) have been integrated into multimodal analgesia strategies. TAPB targets the thoracolumbar nerves that innervate the anterior abdominal wall, effectively reducing postoperative somatic pain following lower abdominal surgeries [7,8]. The bilateral application of TAPB is generally preferred in laparoscopic procedures involving midline incisions or bilateral trocar placements, as it provides broader dermatomal coverage. Nevertheless, unilateral TAPB is still utilized in selected cases, particularly when surgical trauma is confined to the right iliac region, based on clinical judgment and resource considerations [9,10]. Despite these practical considerations, the clinical efficacy of unilateral versus bilateral TAPB under SA in LsA has not been sufficiently studied.

To our knowledge, no prior study has directly compared the analgesic outcomes of bilateral versus unilateral TAPB when combined with SA in a standardized LsA population. This gap in the literature is clinically important, as optimizing block techniques in regional anesthesia may lead to improved patient outcomes, shorter recovery time, and more efficient use of anesthetic agents.

The primary outcome of this study was defined as the time to first analgesic request following surgery. Secondary outcomes included postoperative pain intensity (measured by VAS scores), analgesic consumption, and patient satisfaction scores.

We hypothesize that bilateral TAPB provides superior postoperative analgesia compared to unilateral TAPB when combined with spinal anesthesia in laparoscopic appendectomy.

## 2. Materials and Methods

### 2.1. Study Design and Ethical Approval

This study was conducted as a retrospective chart review using anonymized clinical data from patients who underwent laparoscopic appendectomy between October 2020 and April 2023. As per institutional policy, all patients undergoing surgery sign a standard consent form that includes permission to use anonymized clinical data in future research. Accordingly, the ethics committee approved the use of this pre-existing data without the need for individual consent. The study was approved by the Harran University Clinical Research Ethics Committee (Approval No: HRÜ/23.24.08) and conducted according to Declaration of Helsinki.

### 2.2. Patient Selection

The study included 220 patients aged between 18 and 65, classified as American Society of Anesthesiologists (ASA) physical status I–III. Patients were excluded if they had contraindications to spinal anesthesia or peripheral nerve blocks, known allergies to local anesthetics or opioids, bleeding disorders, or were pregnant or lactating.

### 2.3. Anesthesia Protocol

The same surgical and anesthesiology teams performed all procedures to maintain consistency. During the intraoperative period, all patients received low-dose intravenous midazolam (0.03–0.04 mg/kg) as premedication to reduce anxiety. Additional sedation was administered selectively based on patient discomfort, shoulder pain, or restlessness. Intravenous fentanyl (0.5–1 µg/kg) was used in cases of right shoulder pain or visceral discomfort. Supplemental oxygen at 2–3 L/min via nasal cannula was routinely administered, and all patients were continuously monitored using pulse oximetry, ECG, and non-invasive blood pressure. Spinal anesthesia was administered at the L2–L3 interspace using a 27-gauge pencil-point spinal needle. A total of 3.5 mL of 0.5% hyperbaric bupivacaine combined with 10 µg fentanyl was injected intrathecally. This dosage was selected based on previous studies demonstrating adequate block height for lower abdominal laparoscopic surgeries [11]. To facilitate cephalad spread of the anesthetic and to achieve a target sensory block at the T6 level, a 15° Trendelenburg position was applied for 5–7 min post-injection. Sensory block level was confirmed using pinprick testing. Based on anesthesia records, the mean sensory level achieved was T6 (range: T4–T8).

### 2.4. Group Allocation and TAPB Technique

All laparoscopic appendectomy procedures were performed using a standardized three-port technique, which included one umbilical port (10 mm) and two ancillary ports (5 mm) placed in the right lower quadrant and suprapubic region. The surgical team remained consistent across all procedures. Patients were allocated to bilateral or unilateral TAPB groups based on trocar distribution and incision location. Specifically, patients with central or bilateral port placement—including midline umbilical trocars—received bilateral TAPB (Group 1), whereas patients with right-sided ports only received unilateral TAPB (Group 2). All nerve blocks were administered by an experienced anesthesiologist utilizing a high-frequency linear ultrasound probe with an in-plane needle insertion technique. A total volume of 20 mL local anesthetic mixture—consisting of 5 mL of 2% lidocaine, 10 mL of 0.5% bupivacaine, and 5 mL of normal saline—was deposited in the fascial plane between the internal oblique and transversus abdominis muscles. The success of the TAP block was confirmed by direct visualization of local anesthetic spread between the internal oblique and transversus abdominis muscle layers under ultrasound guidance.

### 2.5. Postoperative Management and Outcome Measures

Patients were transferred to the post-anesthesia care unit (PACU) and monitored until a Modified Aldrete Score of ≥9 was achieved. Standard postoperative analgesia included IV paracetamol (1 g every 6 h) and ketorolac (30 mg every 8 h), with additional analgesics administered as needed.

The primary outcome was the time to first analgesic request. Secondary outcomes included VAS pain scores at 1, 3, 6, 9, 12, 15, 18, and 24 h; total analgesic use in the first 24 h; incidence of right shoulder pain; and patient satisfaction (4-point Likert scale).

The primary outcome, time to first analgesic request, was defined as the time interval between the end of surgery and the first postoperative administration of rescue analgesia. The Visual Analog Scale (VAS) determined this based on standardized nursing assessments. Patients were routinely evaluated every 3 h postoperatively, and analgesia was administered when the VAS score was ≥4. Administration time was recorded in the electronic medical records and extracted retrospectively for analysis.

### 2.6. Statistical Analysis

All statistical procedures were conducted using SPSS v15.0 (SPSS Inc., Chicago, IL, USA). Continuous variables were reported as means and 95% confidence intervals, while categorical data were expressed as frequencies and percentages. The chi-square test was used for categorical comparisons; the Mann–Whitney U test was applied to non-normally distributed continuous variables. Postoperative pain intensity was assessed at eight time points (1, 3, 6, 9, 12, 15, 18, and 24 h). Although this repeated analysis across multiple time points increases the risk of type I error, *p*-values were reported descriptively without formal correction (e.g., Bonferroni adjustment), as the aim was to explore temporal trends in analgesia rather than perform confirmatory testing at each interval. A *p*-value < 0.05 was considered statistically significant. The effect size for the primary outcome (Cohen’s d ≈ 0.5) with a total sample of 220 participants indicated sufficient statistical power.

## 3. Results

The final analysis included 220 patients, evenly divided between the two groups (*n* = 110 per group). Patients in Group 1 underwent bilateral transversus abdominis plane block (TAPB) combined with spinal anesthesia, whereas those in Group 2 received unilateral TAPB under the same neuraxial anesthesia protocol. No significant statistical differences were identified between the groups in terms of demographic and baseline clinical characteristics, including age, gender, height, weight, BMI, ASA score, smoking history, and existing comorbidities (*p* > 0.05 across all parameters; refer to Table 1).

### 3.1. Perioperative Outcomes

There were no significant differences between groups in the incidence of intraoperative hypotension (10.9% in Group 1 vs. 8.2% in Group 2, *p* = 0.491), nausea/vomiting (2.7% vs. 1.8%, *p* = 1.000), or requirement for intraoperative deep sedation due to right shoulder pain (23.6% vs. 25.5%, *p* = 0.754). No conversions to general anesthesia were necessary in either group.

Right-sided shoulder discomfort, commonly associated with pneumoperitoneum during laparoscopic procedures, was reported in 58.2% of patients in Group 1 and 55.5% in Group 2. The difference between the groups was not statistically significant (*p* = 0.683). Among those who experienced shoulder pain, 43.2% required additional intraoperative fentanyl sedation.

### 3.2. Postoperative Analgesia

Patients in Group 1 demonstrated a significantly longer mean time to first analgesic request compared to Group 2 (13.7 h vs. 12.1 h; *p* = 0.001). Additionally, fewer patients in Group 1 required rescue analgesia during the first 24 h postoperatively (13.6% vs. 3.6%; *p* = 0.008). The total number of analgesic doses administered was also significantly lower in the bilateral TAPB group (mean 1.24 vs. 1.32; *p* = 0.010) (Table 2a).

### 3.3. Pain Scores (VAS)

**Figure 1:**
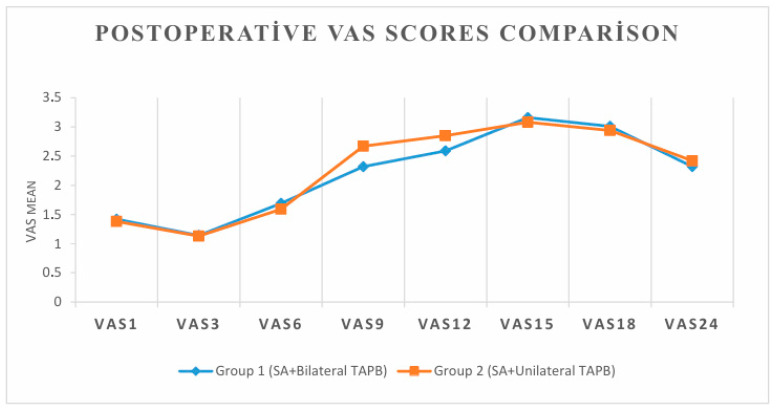
VAS score graph of two groups.

Both groups reported generally low postoperative pain scores. However, VAS scores at the 9th and 12th hours were significantly lower in Group 1 compared to Group 2 (*p* = 0.003 and *p* = 0.039, respectively), suggesting better sustained analgesia with bilateral TAPB. No significant differences were observed at other time points (Table 3, Figure 1).

### 3.4. Patient Satisfaction

Patient satisfaction scores did not differ significantly between the two groups (mean 1.92 vs. 1.85; *p* = 0.084). However, a higher proportion of patients in Group 1 reported being “very satisfied” or “excellent” compared to Group 2 (55.5% vs. 42.7%) (Table 2b).

### 3.5. Shoulder Pain Subgroup Analysis

Patients with shoulder pain had significantly higher VAS scores at 1, 3, and 6 h (*p* < 0.001, *p* < 0.001, *p* = 0.048). Satisfaction scores were numerically lower but not statistically different (*p* = 0.143) (Table 4).

## 4. Discussion

This retrospective study demonstrated that bilateral transversus abdominis plane block (TAPB) combined with spinal anesthesia (SA) provided significantly longer postoperative analgesia and reduced analgesic consumption compared to unilateral TAPB in patients undergoing laparoscopic appendectomy (LsA). Specifically, the mean time to first analgesic requirement was longer in the bilateral TAPB group (13.7 h vs. 12.1 h; *p* = 0.001), and fewer patients in this group required postoperative analgesics (*p* = 0.008). Although overall patient satisfaction scores were similar between groups, a greater proportion of patients in the bilateral TAPB group rated their experience as “very satisfied” or “excellent” (55.5% vs. 42.7%). Moreover, VAS pain scores were significantly lower at the 9th and 12th postoperative hours in patients who received bilateral TAPB, supporting its superior intermediate-term analgesic effect.

The observed superiority of bilateral TAPB over unilateral TAPB can be attributed to the broader dermatomal coverage it provides. Bilateral TAPB more effectively blocks somatic pain pathways from both sides of the abdominal wall, which is particularly relevant in LsA procedures where trocar placement often involves midline and bilateral access points (e.g., umbilical and suprapubic ports). In contrast, unilateral TAPB may be insufficient in covering nociceptive input from contralateral or midline regions, especially when central or multiple incisions are present. The more extensive coverage achieved by bilateral TAPB likely contributes to delayed analgesic need and reduced pain scores after the resolution of spinal anesthesia.

Although GA is more commonly referred to as the routine anesthesia technique for LsA surgery, RA, which has become part of multimodal analgesia, can also be applied in this surgery [12]. Regional anesthesia (RA) offers several notable benefits over general anesthesia (GA), including attenuation of the surgical stress response, elimination of airway manipulation, effective postoperative pain control, and earlier mobilization, which may reduce the risk of venous thromboembolism [5]. Numerous publications have evaluated RA as a viable alternative to GA in laparoscopic procedures. Nevertheless, the use of RA is not without limitations. Potential drawbacks include hypotension resulting from sympathetic blockade, respiratory effects related to the high sensory block levels required, right-sided shoulder discomfort due to diaphragmatic irritation, and the possibility of longer operative times associated with elevated intra-abdominal pressure. In the literature, studies predominantly focus on laparoscopic cholecystectomies performed under CSE [5,6,7,8].

There is minimal literature on LsA surgeries performed under SA [13]. In this context, the present study is significant as it is the only study in LsA patients where TAPB supports SA. A total of 220 patients, divided into two groups of 110 each, underwent SA, which TAPB supported for postoperative analgesia. Although the optimal sensory block level for SA in appendectomy patients is not definitive, a block reaching the T4–T6 level is sufficient for LsA [8,13]. This study achieved all patients’ desired sensory block levels by administering approximately 3.5 mL of local anesthetic at the L2–L3 vertebral level. The most common side effect of SA is hypotension. The incidence of hypotension due to SA in LsA patients was reported by Mane et al. [8] and Jun et al. [13] as 11.5% and 12.5%, respectively. In the present study, hypotension was observed in 10.9% of patients in Group 1 and 8.2% in Group 2, with no statistically significant difference between the groups, aligning with previously published findings. Similarly, both groups were comparable with respect to intraoperative vital parameters, including the incidence of nausea and vomiting, showing no significant variation.

Right shoulder pain, which was frequently reported in both groups (58.2% vs. 55.5%), is a well-known side effect of pneumoperitoneum and diaphragmatic irritation during laparoscopy. Our findings suggest that although bilateral TAPB may not prevent referred shoulder pain, it effectively attenuates somatic pain arising from abdominal wall incisions. The intraoperative Trendelenburg position further contributes to this side effect [8]. A meta-analysis reported that reducing pneumoperitoneum pressure decreases this pain [14]. For optimal visualization during LC, lower pressures, such as 5 to 11 mmHg, are reported to be sufficient [15,16,17]. The incidence of right shoulder pain has been reported to range between 10% and 55.2% [18,19,20,21]. Similar studies identified rates of right shoulder pain alleviated with IV fentanyl as 24.2%, 25%, and 30.8%, respectively [8,13]. In this study, right shoulder pain was detected in 56.8% of patients, with 43.2% of these patients (24.5% of all patients) requiring deep intraoperative sedation (IV fentanyl). None of the patients required conversion to GA for this reason. We attributed the higher incidence of right shoulder pain in this study compared to similar studies to the higher pneumoperitoneum pressure of 14 mmHg used by the surgical team. As mentioned earlier, similar studies in the literature utilized lower pressures. However, the need for deep sedation due to shoulder pain was comparable to that reported in the literature.

Our results are consistent with previous studies that support the analgesic superiority of bilateral TAPB in abdominal and pelvic surgeries. Mukhtar and Singh [22] highlighted the importance of bilateral TAPB in laparoscopic procedures with midline involvement, while Baeriswyl et al. [22] demonstrated its enhanced efficacy over unilateral approaches in terms of postoperative pain control. In laparoscopic cholecystectomy and gynecological surgeries, bilateral TAPB has been associated with reduced opioid consumption and lower VAS scores compared to unilateral applications [10,22,23].

Although the literature on TAPB in laparoscopic appendectomy under SA is limited, our study adds to the growing body of evidence suggesting that combining neuraxial anesthesia with bilateral TAPB yields superior analgesic outcomes. In particular, our findings align with the results of studies on cesarean sections and hernia repairs where bilateral TAPB contributed to prolonged analgesia and greater patient satisfaction [15,17,23].

As with other surgeries, neuraxial and RA techniques provide postoperative analgesia for a specific period. A study comparing GA and RA techniques in laparoscopic surgeries showed that 50 LsC patients in the SA group required less analgesia postoperatively and had significantly lower pain scores [24]. In another study comparing GA and thoracic SA in laparoscopic surgeries, thoracic SA was associated with more effective postoperative analgesia, easier surgical conditions, and significantly higher patient satisfaction [25]. Another study involving 60 patients comparing SA and GA reported better postoperative analgesia and higher patient satisfaction with SA [26]. However, unilateral TAP block (TAPB) is more effective in cases where lateralized pain is predominant. In laparoscopic surgeries, particularly for unilateral incisions or surgical interventions confined to one side, unilateral TAPB may be sufficient. However, in cases involving bilateral surgical trauma or trocar placement on both sides, bilateral TAPB may provide more effective analgesia [22]. It has been observed that patients receiving bilateral TAPB have lower postoperative pain scores compared to those receiving unilateral TAPB. In particular, bilateral block application has been shown to enhance analgesic efficacy in patients with midline incisions [23]. In this study, since trocars were predominantly placed in the correct quadrant, Group 2 patients received unilateral TAPB. In contrast, Group 1 patients underwent bilateral TAPB due to the midline placement of the umbilical trocar. The groups were then compared in terms of postoperative analgesia and patient satisfaction. In this regard, this study supports the findings of previous literature.

In light of this information, SA was applied to both groups in our study, with bilateral TAPB performed in Group 1 and unilateral TAPB in Group 2. The most significant finding of this study was that the SA + TAPB combination provided prolonged and pain-free postoperative periods. Postoperative analgesia lasted an average of 13.7 h in Group 1 and 12.1 h in Group 2, with a significant difference between the groups (*p* = 0.001). Although Group 1 had a longer postoperative analgesic duration, the duration was sufficient in both groups.

Additionally, the requirement for postoperative analgesics was significantly lower in Group 1 compared to Group 2 (*p* = 0.008). The number of patients needing analgesia within the first 24 h after surgery was also markedly reduced in Group 1 (*p* = 0.010). Although overall patient satisfaction was high in both groups, the difference between them did not reach statistical significance (*p* = 0.084). However, when evaluated using the Likert score, the proportion of patients reporting “very satisfied/excellent” was higher in Group 1 (55.5% vs. 42.7%). Thus, this study supports and confirms the findings in the literature regarding the positive effects of RA on patient satisfaction [4,15,17].

In studies analyzing VAS scores in laparoscopic cases performed under neuroaxial anesthesia, a study comparing SA and GA reported significantly lower VAS scores at 4, 8, 12, and 24 h in the SA group (*p* = 0.0001) [26]. Another study comparing SA and GA found lower VAS scores and significantly reduced analgesic requirements in the SA group at PACU admission (*p* < 0.05) [24]. In another study, VAS scores at 1, 2, 4, and 8 h postoperatively were significantly lower in the SA group compared to GA, but no significant difference was observed at 24 h [27]. In a study comparing postoperative VAS scores between GA and CSE anesthesia, VAS scores in the CSE anesthesia group were significantly lower immediately after surgery and at the 12th and 24th hours (*p* < 0.001). However, no significant difference was observed in VAS scores at the 6th hour (*p* = 0.274) [28]. In our study, since SA was applied to both groups, overall VAS scores were low throughout all time intervals. However, when comparing VAS scores between the groups due to bilateral versus unilateral TAPB, no significant differences were observed at the 1st, 3rd, 6th, 15th, 18th, and 24th hours. However, significant differences were noted at the 9th (*p* = 0.003) and 12th hours (*p* = 0.039), with higher VAS scores observed in Group 2 during these times. This was attributed to more pronounced pain on the side without TAPB after the effect of SA diminished. Similar to the literature, bilateral TAPB applications have been reported to provide longer-lasting and more effective analgesia than unilateral applications [4,14,15]. This finding supports our assertion that if a plane block is to be performed for postoperative analgesia in neuraxial anesthesia cases, it should be performed bilaterally.

Additionally, when comparing VAS scores between patients with and without shoulder pain, significant differences were observed in the 1st (*p* < 0.001), 3rd (*p* < 0.001), and 6th hours (*p* = 0.048), but no significant differences were noted at later time intervals. This confirms that the effect of pneumoperitoneum persists for a specific postoperative time frame. Therefore, as emphasized in the literature [15,16,17], laparoscopic surgery performed at lower pneumoperitoneum pressures can positively impact postoperative analgesic requirements and patient satisfaction.

### Limitations

This study has several important limitations. First, its retrospective observational design inherently carries a risk of selection bias, and the absence of randomization limits the ability to draw causal inferences. Although baseline demographic characteristics were comparable between the two groups, no propensity score matching or multivariable adjustments were employed to control for potential confounding variables. Second, the decision to perform unilateral or bilateral TAPB was based on clinical judgment rather than a standardized protocol. Furthermore, the specific trocar insertion sites were not consistently documented in the patient records, limiting the ability to retrospectively verify the rationale for block allocation and representing an additional methodological limitation. Third, the use of repeated VAS assessments at multiple postoperative time points increases the risk of type I error. Since no correction for multiple comparisons (e.g., Bonferroni adjustment) was applied, isolated statistically significant differences should be interpreted with caution. Nevertheless, the findings remain consistent with the primary outcome—time to first analgesic request—thereby supporting the robustness of the main conclusion.

## 5. Conclusions

This study demonstrates that adding bilateral transversus abdominis plane block (TAPB) to spinal anesthesia provides superior postoperative analgesia compared to unilateral TAPB in patients undergoing laparoscopic appendectomy. Bilateral TAPB was associated with longer pain-free intervals, reduced need for rescue analgesia, and improved intermediate pain scores without increased complications or adverse effects. These findings support the use of bilateral TAPB as a more effective analgesic strategy in laparoscopic abdominal surgery, particularly in cases involving midline or multiple trocar placements. Future prospective, randomized studies are warranted to validate these results further and explore optimal combinations of regional techniques in ambulatory surgical settings.

### 5.1. What Is Known

Spinal anesthesia is an effective alternative to general anesthesia for laparoscopic abdominal surgeries, offering better postoperative analgesia and fewer complications.

Transversus abdominis plane block (TAPB) enhances postoperative analgesia in abdominal procedures and is often applied bilaterally for wider coverage.

### 5.2. What Is New

This study is the first to compare unilateral and bilateral TAPB combined with spinal anesthesia in laparoscopic appendectomy.

Bilateral TAPB significantly prolonged the time to first analgesic requirement and reduced analgesic consumption compared to unilateral TAPB.

Bilateral TAPB led to better intermediate pain scores and higher patient satisfaction, without increasing complications.

## Figures and Tables

**Table 1:** Demographic characteristics of the study groups.

Variable		Group 1 (SA + Bilateral TAPB)	Group 2 (SA + Unilateral TAPB)	*p*-Value
Gender *n* (%)	Male	67 (60.9)	63 (57.3)	0.583 *
	Female	43 (39.1)	47 (42.7)	
Age mean (95% CI)		37.2 (34.3–40)	33.8 (31.4–36.2)	0.224 ^†^
Height means cm (95% CI)		168.5 (166.8–170.3)	169 (167.4–170.6)	0.797 ^†^
Weight means kg (95% CI)		79.9 (76.9–83)	80 (76.9–83.2)	0.467 ^†^
BMI mean (95% CI)		28.3 (27.1–29.4)	28.2 (27–29.4)	0.367 ^†^
ASA *n* (%)	ASA I	53 (48.2)	59 (53.6)	0.717
	ASA II	42 (38.2)	38 (34.5)	
	ASA III	15 (13.6)	13 (11.8)	
Comorbidities *n* (%)	None	77 (70.0)	83 (75.5)	0.364
	Present	33 (30.0)	27 (24.5)	
Smoking *n* (%)	Non-smoker	75 (68.2)	78 (70.9)	0.66
	Smoker	35 (31.8)	32 (29.1)	

* Chi-Square Test; ^†^ Mann–Whitney U Test; TAPB: Transverse Abdominal Plane Block; BMI: Body Mass Index; ASA: American Society of Anesthesiologists; CI: Confidence Interval.

**Table 2:** (a) Perioperative findings and postoperative analgesia. (b) Patient satisfaction based on the Likert scale.

Variable		Group 1 (SA + Bilateral TAPB)	Group 2 (SA + Unilateral TAPB)	*p*-Value
(a)				
Right Shoulder Pain *n* (%)	None	46 (41.8)	49 (44.5)	0.683 *
	Present	64 (58.2)	61 (55.5)	
Hypotension *n* (%)	None	98 (89.1)	101 (91.8)	0.491 *
	Present	12 (10.9)	9 (8.2)	
Nausea/Vomiting *n* (%)	None	107 (97.3)	108 (98.2)	1.000 *
	Present	3 (2.7)	2 (1.8)	
Deep sedation during surgery *n* (%)	Not Required	84 (76.4)	82 (74.5)	0.754 *
	Required	26 (23.6)	28 (25.5)	
Operation Time (minute)		34.3 ± 8.7	32.9 ± 8.5	0.229 ^†^
Satisfaction mean (95% CI min-maks)		1.92 (1.86–1.97)	1.85 (1.78–1.92)	0.084 ^†^
Postoperative Analgesia Needed *n* (%)	None	15 (13.6)	4 (3.6)	0.008 *
	Present	95 (86.4)	106 (96.4)	
Time to First Analgesic (hours) mean (95% CI min-maks)		13.7 (12.9–14.6)	12.1 (11.4–12.8)	0.001 ^†^
Analgesic Doses in First 24 h mean (95% CI min-maks)		1.24 (1.14–1.34)	1.32 (1.22–1.42)	0.010 ^†^
(b)				
Satisfaction Level *n* (%)	Slightly Satisfied	1 (0.9)	0 (0.0)	0.07
	Neutral	7 (6.4)	15 (13.6)	
	Satisfied	41(37.3)	48 (43.6)	
	Very Satisfied/Excellent	61 (55.5)	47 (42.7)	

* Chi-Square Test; ^†^ Mann–Whitney U Test; TAPB: Transverse Abdominal Plane Block; CI: Confidence Interval; SA: Spinal Anesthesia.

**Table 3:** Postoperative VAS scores comparison.

Time	Group 1 (SA + Bilateral TAPB)	Group 2 (SA + Unilateral TAPB)	*p*-Value *
VAS1 mean (95% CI min-maks)	1.42 (1.26–1.58)	1.38 (1.24–1.52)	0.902
VAS3 mean (95% CI min-maks)	1.14 (1.06–1.21)	1.13 (1.06–1.21)	0.981
VAS6 mean (95% CI min-maks)	1.69 (1.48–1.91)	1.59 (1.40–1.78)	0.941
VAS9 mean (95% CI min-maks)	2.32 (2.06–2.57)	2.67 (2.42–2.92)	0.003
VAS12 mean (95% CI min-maks)	2.59 (2.34–2.84)	2.85 (2.58–3.12)	0.039
VAS15 mean (95% CI min-maks)	3.16 (2.90–3.41)	3.08 (2.84–3.31)	0.481
VAS18 mean (95% CI min-maks)	3.01 (2.76–3.27)	2.94 (2.70–3.18)	0.905
VAS24 mean (95% CI min-maks)	2.32 (2.17–2.46)	2.42 (2.29–2.56)	0.172

* Mann–Whitney U Test; TAPB: Transverse Abdominal Plane Block; SA: Spinal Anesthesia.

**Table 4:** Comparison of patients with and without shoulder pain.

Variable	Right Shoulder Pain			*p*-Value
		None	Present	
Intraoperative Deep Sedation *n* (%)	Not Required	95 (100)	71 (56.8)	<0.001 *
	Required	0 (0.0)	54 (43.2)	
VAS1 mean (95% CI min-max)		1 (1–1)	1.70 (1.53–1.86)	<0.001 ^†^
VAS3 mean (95% CI min-max)		1 (1–1)	1.23 (1.15–1.32)	<0.001 ^†^
VAS6 mean (95% CI min-max)		1.40 (1.25–1.54)	1.83 (1.61–2.05)	0.048 ^†^
VAS9 mean (95% CI min-max)		2.36 (2.12–2.60)	2.61 (2.36–2.86)	0.233 ^†^
VAS12 mean (95% CI min-max)		2.69 (2.39–2.98)	2.76 (2.52–2.99)	0.489 ^†^
VAS15 mean (95% CI min-max)		3.01 (2.75–3.28)	3.19 (2.96–3.42)	0.270 ^†^
VAS18 mean (95% CI min-max)		2.98 (2.69–3.26)	2.97 (2.76–3.19)	0.719 ^†^
VAS24 mean (95% CI min-max)		2.41 (2.27–2.54)	2.35 (2.21–2.49)	0.393 ^†^
Satisfaction mean (95% CI min-max)		1.92 (1.86–1.98)	1.85 (1.79–1.92)	0.143 ^†^
Postoperative Analgesia *n* (%)		86 (90.5)	115 (92.0)	0.700 *
Time to First Analgesic (95% CI min-max)		13.23 (12.5–13.97)	12.61 (11.82–13.4)	0.366 ^†^
Analgesic Doses in First 24 Hours (95% CI min-max)		1.27 (1.17–1.37)	1.30 (1.2–1.39)	0.655 ^†^

* Chi-Square Test; ^†^ Mann–Whitney U Test; VAS: Visual Analog Scale.

## Data Availability

The data presented in this study are available on request from the corresponding author due to (specify the reason for the restriction).

## References

[1] Li X., Zhang J., Sang L., Zhang W., Chu Z., Li X., Liu Y. (2010). Laparoscopic versus conventional appendectomy—A meta-analysis of randomized controlled trials. BMC Gastroenterol..

[2] Ozgün H., Kurt M.N., Kurt I., Cevikel M.H. (2002). Comparison of local, spinal, and general anesthesia for inguinal herniorrhaphy. Eur. J. Surg..

[3] Sinha R., Gurwara A.K., Gupta S.C. (2008). Laparoscopic total extraperitoneal inguinal hernia repair under spinal anesthesia: A study of 480 patients. J. Laparoendosc. Adv. Surg. Tech. A.

[4] Bajwa S.J.S., Kulshrestha A. (2016). Anaesthesia for laparoscopic surgery: General vs regional anesthesia. J. Minimal Access Surg..

[5] Lal P., Philips P., Saxena K.N., Kajla R.K., Chander J., Ramteke V.K. (2007). Laparoscopic total extraperitoneal (TEP) inguinal hernia repair under epidural anesthesia: A detailed evaluation. Surg. Endosc..

[6] Hamad M.A., El-Khattary O.A. (2003). Laparoscopic cholecystectomy under spinal anesthesia with nitrous oxide pneumoperitoneum: A feasibility study. Surg. Endosc..

[7] van Zundert A.A.J., Stultiens G., Jakimowicz J.J., Peek D., van der Ham W.G.J.M., Korsten H.H.M., Wildsmith J.A.W. (2007). Laparoscopic cholecystectomy under segmental thoracic spinal anesthesia: A feasibility study. Br. J. Anaesth..

[8] Lee J.H., Huh J., Kim D.K., Gil J.R., Min S.W., Han S.S. (2010). Laparoscopic cholecystectomy under epidural anesthesia: A clinical feasibility study. Korean J. Anesthesiol..

[9] Ozciftci S., Sahiner Y., Sahiner İ.T., Akkaya T. (2022). Is Right Unilateral Transversus Abdominis Plane (TAP) Block Successful in Postoperative Analgesia in Laparoscopic Cholecystectomy?. Int. J. Clin. Pract..

[10] Rahimzadeh P., Faiz S.H.R., Latifi-Naibin K., Alimian M. (2022). A Comparison of the effect of preemptive versus postoperative use of ultrasound-guided bilateral transversus abdominis plane (TAP) block on pain relief after laparoscopic cholecystectomy. Sci. Rep..

[11] Chung C.-J., Choi S.-R., Yeo K.-H., Park H.-S., Lee S.-I., Chin Y.-J. (2000). Hyperbaric spinal anesthesia with 0.5% bupivacaine: A comparison of doses. Reg. Anesth. Pain Med..

[12] Collins L.M., Vaghadia H. (2001). Regional anesthesia for laparoscopy. Anesthesiol. Clin. N. Am..

[13] Jun G.W., Kim M.S., Yang H.J., Sung T.Y., Park D.H., Cho C.K., Kwon H.U., Kang P.S., Moon J.I. (2014). Laparoscopic appendectomy under spinal anesthesia with dexmedetomidine infusion. Korean J. Anesthesiol..

[14] Gurusamy K.S., Samraj K., Davidson B.R. (2009). Low-pressure versus standard pressure pneumoperitoneum in laparoscopic cholecystectomy. Cochrane Database Syst. Rev..

[15] Gerges F.J., Kanazi G.E., Jabbour-Khoury S.I. (2006). Anesthesia for laparoscopy: A review. J. Clin. Anesth..

[16] Spivak H., Nudelman I., Fuco V., Rubin M., Raz P., Peri A., Lelcuk S., Eidelman L.A. (1999). Laparoscopic extraperitoneal inguinal hernia repair with spinal anesthesia and nitrous oxide insufflation. Surg. Endosc..

[17] Sarli L., Costi R., San Sebastiano G., Trivelli M., Roncoroni L. (2000). Prospective randomized trial of low-pressure pneumoperitoneum for reduction of shoulder-tip pain following laparoscopy. Br. J. Surg..

[18] Tzovaras G., Fafoulakis F., Pratsas K., Georgopoulou S., Stamatiou G., Hatzitheofilou C. (2008). Spinal vs. general anesthesia for laparoscopic cholecystectomy. Interim analysis of a controlled, randomized trial. Arch. Surg..

[19] Sinha R., Gurwara A.K., Gupta S.C. (2009). Laparoscopic cholecystectomy under spinal anesthesia: A study of 3492 patients. J. Laparoendosc. Adv. Surg. Tech. A.

[20] Tiwari S., Chauhan A., Chatterjee P., Alam M.T. (2013). Laparoscopic cholecystectomy under spinal anesthesia: A prospective, randomized study. J. Minimal Access Surg..

[21] Goyal S., Goyal S., Singla S. (2012). Laparoscopic Cholecystectomy Under Spinal Anesthesia with Low-Pressure Pneumoperitoneum—Prospective Study of 150 Cases. Arch. Clin. Exp. Surg..

[22] Mukhtar K., Singh S. (2009). Transversus abdominis plane block for laparoscopic surgery. Br. J. Anaesth..

[23] Baeriswyl M., Zeiter F., Piubellini D., Kirkham K.R., Albrecht E. (2018). The analgesic efficacy of transverse abdominis plane block versus epidural analgesia: A systematic review with meta-analysis. Medicine.

[24] Turkstani A., Ibraheim O., Khairy G., Alseif A., Khalil N. (2009). Spinal versus general anesthesia for laparoscopic cholecystectomy: A cost-effectiveness and side effects study. Anaesth. Pain Intensive Care.

[25] Ellakany M. (2013). Comparative study between general and thoracic spinal anesthesia for laparoscopic cholecystectomy. Egypt. J. Anaesth..

[26] Mehta P.J., Chavda H.R., Wadhwana A.P., Porecha M.M. (2010). Comparative analysis of spinal versus general anesthesia for laparoscopic cholecystectomy: A controlled, prospective, randomized trial. Anesth. Essays Res..

[27] Kalaivani V., Pujari V.S., Sreevathsa M.R., Hiremath B.V., Bevinaguddaiah Y. (2014). Laparoscopic Cholecystectomy Under Spinal Anaesthesia vs. General Anaesthesia: A Prospective Randomised Study. J. Clin. Diagn. Res..

[28] Erdem V.M., Donmez T., Uzman S., Ferahman S., Hatipoglu E., Sunamak O. (2018). Spinal/epidural block as an alternative to general anesthesia for laparoscopic appendectomy: A prospective randomized clinical study. Videosurg. Other Miniinvasive Tech..

